# DermViT: Diagnosis-Guided Vision Transformer for Robust and Efficient Skin Lesion Classification

**DOI:** 10.3390/bioengineering12040421

**Published:** 2025-04-16

**Authors:** Xuejun Zhang, Yehui Liu, Ganxin Ouyang, Wenkang Chen, Aobo Xu, Takeshi Hara, Xiangrong Zhou, Dongbo Wu

**Affiliations:** 1 School of Computer, Electronics and Information, Guangxi University, Nanning 530004, China; xjzhang@gxu.edu.cn (X.Z.);; 2 Department of Electrical, Electronic and Computer Engineering, Gifu University, Gifu 501-1193, Japan; 3 Department of General Surgery, The Fourth Affiliated Hospital of Guangxi Medical University, Liuzhou 545000, China; 4 Department of Gastrointestinal, Metabolic and Bariatric Surgery, Ruikang Hospital Affiliated to Guangxi University of Chinese Medicine, Nanning 530004, China

**Keywords:** skin cancer, skin lesion classification, transformer, dermoscopic image analysis

## Abstract

Early diagnosis of skin cancer can significantly improve patient survival. Currently, skin lesion classification faces challenges such as lesion–background semantic entanglement, high intra-class variability, artifactual interference, and more, while existing classification models lack modeling of physicians’ diagnostic paradigms. To this end, we propose DermViT, a medically driven deep learning architecture that addresses the above issues through a medically-inspired modular design. DermViT consists of three main modules: (1) Dermoscopic Context Pyramid (DCP), which mimics the multi-scale observation process of pathological diagnosis to adapt to the high intraclass variability of lesions such as melanoma, then extract stable and consistent data at different scales; (2) Dermoscopic Hierarchical Attention (DHA), which can reduce computational complexity while realizing intelligent focusing on lesion areas through a coarse screening–fine inspection mechanism; (3). Dermoscopic Feature Gate (DFG), which simulates the observation–verification operation of doctors through a convolutional gating mechanism and effectively suppresses semantic leakage of artifact regions. Our experimental results show that DermViT significantly outperforms existing methods in terms of classification accuracy (86.12%, a 7.8% improvement over ViT-Base) and number of parameters (40% less than ViT-Base) on the ISIC2018 and ISIC2019 datasets. Our visualization results further validate DermViT’s ability to locate lesions under interference conditions. By introducing a modular design that mimics a physician’s observation mode, DermViT achieves more logical feature extraction and decision-making processes for medical diagnosis, providing an efficient and reliable solution for dermoscopic image analysis.

## 1. Introduction

Early diagnosis of skin cancer is crucial for improving patient prognosis. The five-year survival rate for malignant melanoma can reach 99% with early detection, but declines sharply to 23% when diagnosed at advanced stages [1]. Dermoscopy [2], which provides structural information on the epidermis, has become an important non-invasive diagnostic tool [3]. However, this technique is highly dependent on physicians’ expertise and subjective assessment, resulting in a considerable risk of misdiagnosis [4]. Therefore, there is an urgent need to develop efficient and reliable computer-aided diagnostic systems [5].

**Figure 1:**
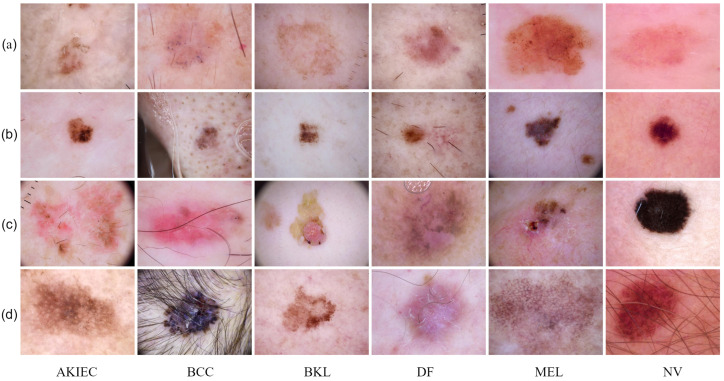
Examples of skin lesions from ISIC2018 dataset; each column represents the same type of disease. One challenge to the classification of skin lesions is high intra-class variation. Columns (**a**,**b**) show examples of high inter-class similarity, while columns (**c**,**d**) show examples of hair and other artifacts.

As illustrated in Figure 1, automatic classification of skin diseases faces significant challenges, including high inter-class similarity, high intra-class variability, and artifacts such as hair and air bubbles. Early research was primarily based on traditional methods that combined handcrafted feature extraction and machine learning [6]. For instance, Celebi et al. [7] proposed an image segmentation strategy based on Euclidean distance transformation, while Abbas et al. [8] constructed color–texture feature models in color space. Although these methods achieved some success on limited datasets, they were constrained by inherent limitations such as complex feature engineering and weak generalization capabilities.

The advent of convolutional neural networks (CNNs) revolutionized skin lesion classification [9]. Esteva et al. [10] showed that CNNs could match dermatologists in diagnostic accuracy. Harangi et al. [11] achieved enhanced classification robustness through model ensemble strategies, while Aldhyani et al. [12] balanced efficiency and accuracy through a dynamic kernel design. However, their local receptive field makes CNNs insufficient for modeling the global contextual relationships of lesions, which limits their ability to handle diffuse lesions [13]. To address this issue, researchers have incorporated attention mechanisms to enhance classification performance. For instance, Gessert et al. [14] proposed a patch-based attention mechanism combined with diagnosis-guided loss weighting to improve the focus on hard-to-classify cases and strengthen global feature modeling. More recently, Yu et al. [15] have demonstrated the effectiveness of cross-attention mechanisms for multimodal feature integration, achieving 91% accuracy in melanoma classification.

The success of the transformer architecture [16] in natural language processing has provided a new paradigm for visual tasks. The vision transformer (ViT) architecture [17] were the first to validate the visual representation capability of pure transformer models on ImageNet classification tasks by partitioning images into sequential patches and modeling global dependencies through self-attention mechanisms. This breakthrough has facilitated its application in medical imaging. For instance, Soumyya et al. [18] introduced a soft attention mechanism to improve ViT’s focus on lesion regions, resulting in reduced dependency on global features and enhanced classification accuracy. Zhao et al. [13] introduced a fully transformer-based FTN network that achieved efficient feature learning, while Li et al. [19] designed a CNN–transformer dual-branch model that eliminated semantic discrepancies between the two branches using the BC-FCU module, raising the multi-class classification accuracy to 87.6%. These and similar advancements [20,21,22] demonstrate that transformer-based models offer significant advantages in addressing the locality limitations of CNNs and capturing long-range dependencies.

However, the original ViT design faces significant challenges in medical imaging. First, the computational complexity of the global self-attention mechanism scales quadratically with image resolution [23]. This leads to substantial computational overhead for high-resolution dermoscopic images, which typically exceed 1024 × 1024 pixels [24]. Second, ViT lacks incorporation of domain-specific medical knowledge; dermatologists rely on a multiscale observation paradigm (i.e., first identifying regions of interest at low magnification and then refining the diagnosis at high magnification) and an attention-focusing mechanism (i.e., initially filtering out irrelevant regions before analyzing lesion features in detail), whereas ViT’s uniform allocation of attention does not align with this diagnostic logic [25]. More critically, ViT treats all visual tokens equally during feature propagation [26], which allows irrelevant artifacts such as hair or bubbles to introduce noise into the deep semantic representations. The small sample size typical of medical datasets (e.g., ISIC2018 contains only 10,015 images) further increases the likelihood of overfitting to spurious features. To address these issues, we propose DermViT, which mitigates the inherent limitations of ViT through a structural redesign inspired by medical diagnostic principles. DermViT consists of the following key modules:

1. Dermoscopic Hierarchical Attention (DHA): DHA mimics the attention-shifting mechanism employed by dermatologists, enabling efficient allocation of computational resources through a two-stage routing mechanism. In the first stage, DHA computes region-level attention weights in order to rapidly discard diagnostically irrelevant background regions. In the second stage, fine-grained token-level attention is applied exclusively within the retained regions, concentrating on dermatologically relevant local features. This hierarchical coarse-to-fine strategy reduces computational overhead while enhancing the sensitivity of detection for subtle lesions.
2. Dermoscopic Context Pyramid (DCP): Inspired by the multi-magnification collaborative mechanism in pathological diagnosis, DCP adapts to the high intra-class variability of lesions such as melanoma through cross-scale feature fusion.
3. Dermoscopic Feature Gate (DFG): Inspired by the observation–verification cycle in clinical diagnosis, DFG suppresses semantic noise through channel decoupling and dynamic gating. It decouples input features into a primary pathway for morphological analysis and an auxiliary pathway for detail verification. The primary pathway captures local context (e.g., pigment texture), while the auxiliary pathway calibrates response intensity through channel-wise multiplication. This mechanism emulates the cognitive process of dermatologists, who first observe globally before zooming in to verify local details, effectively attenuating high-frequency artifacts (e.g., hair noise) that may interfere with diagnosis.

The experimental results demonstrate that DermViT achieves strong classification capability without pretraining. In the presence of challenging background artifacts, its attention heatmaps strongly align with ground = truth lesion annotations, surpassing counterparts based on both CNN [27,28,29,30] and transformer [17,19,31] architectures. The proposed model outperforms existing SOTA methods in classification accuracy while substantially enhancing efficiency and resource utilization, offering an optimized and dependable framework for automated skin cancer classification.

## 2. Materials and Methods

This section introduces the proposed methodology. We first describe the key modules of the model, then elaborate on the overall framework in the final section to present the overall design concept of the approach.

### 2.1. Dermoscopic Hierarchical Attention

**Figure 2:**
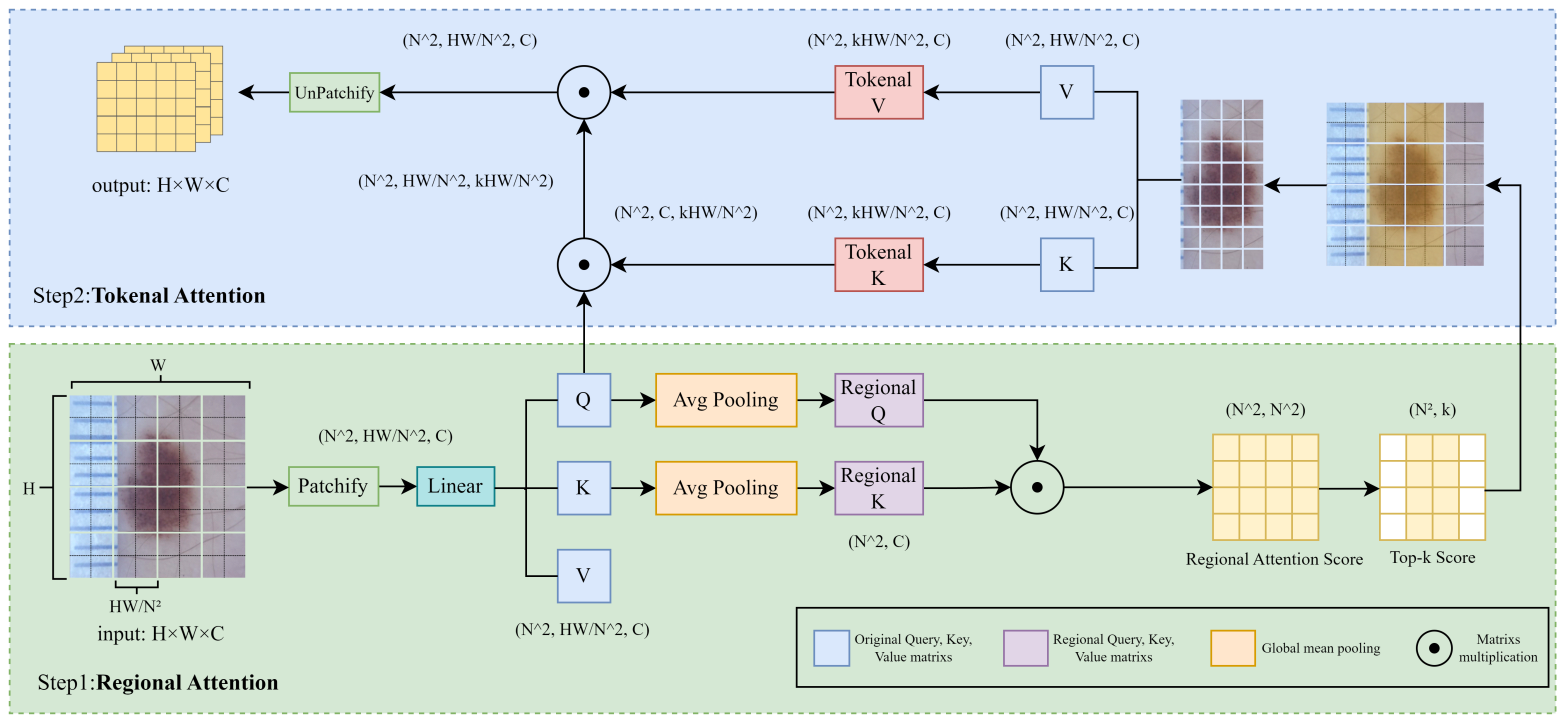
Dermoscopic Hierarchical Attention: The DHA module’s hierarchical attention involves (1) region-level coarse screening for global relevance followed by (2) fine-grained attention focusing on the top-K regions. This design filters obvious background noise (e.g., rulers) while improving both efficiency and precision.

The traditional global self-attention mechanism treats all regions uniformly, resulting in inefficient resource utilization and feature contamination [32]. To address this issue, DHA draws inspiration from sparse attention [23,24,26] and employs a hierarchical self-attention mechanism to mimic the hierarchical diagnostic process of dermatologists through a two-stage process consisting of coarse region filtering followed by fine-grained pixel inspection. The specific process is shown in Figure 2.

Regional Coarse Screening for Filtering Out Irrelevant Background. The input feature map is first divided into N regions through a reshape operation. After reshaping, the image undergoes linear mapping to generate the corresponding original matrices for the query, key, and value, i.e., $Q,K,V\in R^{N\times \frac{HW}{N}\times d}$, where *N* is the number of regions, HW is the size of the feature map, and *d* is the feature dimension. To quickly filter key regions, DHA applies global average pooling (GAP) to *Q* and *K* to obtain region-level queries and keys:

(1) $$Q^{r}=\mathrm{GAP}\left(Q\right)\in R^{N\times d},\;K^{r}=\mathrm{GAP}\left(K\right)\in R^{N\times d}.$$

Based on $Q^{r}$ and $K^{r}$, the regional similarity score matrix is computed as follows:

(2) $$S=Q^{r}\cdot {\left(K^{r}\right)}^{T}\in R^{N\times N}.$$

DHA employs a selective screening mechanism that retains only the top-*k* most relevant regions:

(3) Rtop−k=Topk(S)⊂1,2,…,N.

By discarding the N−k least relevant regions, this strategy significantly reduces computational complexity while effectively suppressing background artifacts such as hair and measurement scales.

Token-Level Refinement Focusing on Lesion Characteristics. Among the *k* candidate regions identified in the previous step, DHA extracts the token-level key ($K^{t}$) and value ($V^{t}$) matrices from K,V based on the similarity score matrix S, then applies fine-grained self-attention as follows:

(4) $$\mathrm{Attention}\left(Q,K^{t},V^{t}\right)=\mathrm{Softmax}\left(\frac{Q\cdot {\left(K^{t}\right)}^{T}}{\sqrt{d_{k}}}\right)\cdot V^{t}.$$

In the above equation, Q represents the original query matrix, while $K^{t}$ and $V^{t}$ respectively correspond to the key and value matrices of the selected candidate regions. This stage simulates the detailed examination process of a dermatologist using magnification, which helps to ensure precise focus on subtle lesion features. After computing the self-attention, the feature map is restored to its original size of $Y\in R^{N\times W\times C}$ before being passed to the next stage.

### 2.2. Dermoscopic Feature Gate

**Figure 3:**
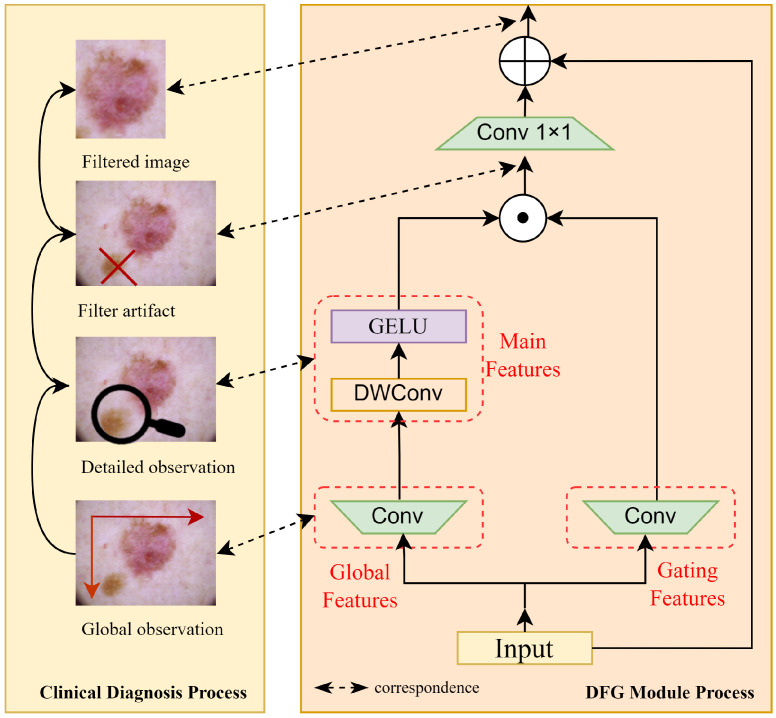
Dermoscopic Feature Gate: The left figure shows the diagnostic logic of a dermatologist; the right figure shows how the design of the DFG module is based on this logic, simulating the process of filtering out artifacts during a doctor’s diagnostic process.

In dermoscopic images, lesion regions are often highly coupled with artifacts such as hairs and blisters, making it difficult for traditional feature propagation mechanisms to effectively suppress the semantic leakage of artifact-related features. To address this issue, the proposed Dermoscopic Feature Gate (DFG) is inspired by the “observation–verification” diagnostic loop used by dermatologists. Specifically, clinicians first perform a global observation to locate suspicious areas, followed by a detailed examination under magnification to verify pathological features, then finally apply their clinical expertise to filter out irrelevant information. As shown in Figure 3, DFG formalizes this process into a learnable feature operation through a dual-path gated convolutional mechanism, which simulates the diagnostic logic of dermatologists in feature space to significantly increase the model’s robustness against interference from artifacts. Given an input feature map $X\in R^{H\times W\times C}$, the DFG module operates as shown below.

Global Observation (Feature Decoupling). The input feature map $X\in R^{C\times H\times W}$ is first processed through a hidden layer to increase feature complexity. This step simulates a dermatologist’s initial global observation, where the clinician scans the entire image to identify potential lesion regions. The resulting feature map $Z\in R^{2C\times H\times W}$ is then split into two parts, $X_{1}$ and V, respectively representing the global features and gating features:

(5) $$X_{1},V=\mathrm{split}\left({\mathrm{Conv}}_{2\times C}\left(X\right)\right)\in R^{2C\times H\times W}.$$

Detailed Inspection (Spatial Feature Extraction). Next, $X_{1}$ is processed through a 3×3 depthwise separable convolution, which enhances spatial feature extraction without significantly increasing the parameter count. This step mimics a dermatologist’s detailed inspection under magnification, where fine-grained features such as irregular borders and color heterogeneity are analyzed. The output $X_{2}$ captures the refined spatial features:

(6) $$X_{2}=\mathrm{ReLU}\left({\mathrm{Conv}}_{\mathrm{depthwise}}\left(X_{1}\right)\right)\in R^{2C\times H\times W}.$$

Clinical Decision (Feature Filtering). Finally, $X_{2}$ is multiplied by the gating signal V to dynamically calibrate the response strength of the observing branch. This helps to ensure that only information relevant to the lesion is enhanced while effectively suppressing background noise, resulting in a calibrated feature. This step emulates the clinician’s decision-making process, where irrelevant information (e.g., hairs, rulers) is filtered out based on clinical experience and only diagnostically relevant features are retained:

(7) $$Y=\mathrm{Dropout}\left({\mathrm{Conv}}_{1\times 1}\left(\left(X_{2}⊙V\right)\right)\right)\in R^{C\times H\times W}.$$

By integrating these steps, the DFG module effectively mimics the dermatologist’s diagnostic workflow, enabling the model to dynamically focus on lesion-specific features while suppressing interference from artifacts. Furthermore, this mechanism significantly reduces computational complexity, achieving more efficient lesion feature extraction compared to traditional feature modeling approaches.

### 2.3. Dermoscopic Context Pyramid

ViT exhibits an inherent contradiction between its global unified attention mechanism and the multi-scale observational paradigm of dermatological diagnosis. Clinicians typically first localize lesion regions at low magnification, then switch to high magnification to analyze the detailed features. In contrast, ViT’s fixed-resolution processing mode fails to accommodate this dynamic cognitive process. Additionally, ViT’s neglect of cross-scale contextual information results in blurred lesion boundaries, severely limiting diagnostic accuracy. The Dermoscopic Contextual Perception (DCP) module addresses this limitation by structurally modeling a multi-magnification collaborative mechanism that simulates a clinician’s cognitive process of cross-scale feature comparison. This provides multi-granularity semantic support for the subsequent DHA mechanism.

**Figure 4:**
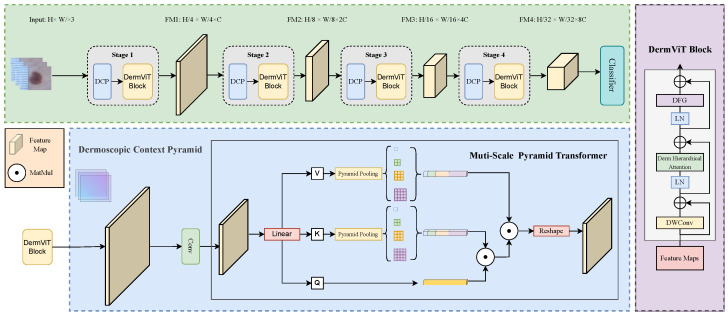
DermViT: Our proposed multi-stage framework. Each stage contains the following modules: (1) DCP for multi-scale feature-preserving downsampling; (2) DHA with two-stage attention (region filtering and token refinement) to suppress irrelevant background; and (3) DFG for imaging artifact removal, enabling robust lesion classification.

As shown in Figure 4, the DCP consists of a spatial compressor with a multiscale pyramid transformer (MPT). For simplicity, we only draw the key attention schema in the MPT shown in Figure 4. The computational flow is closely aligned with the dermoscopic diagnostic criteria:

Low-Magnification Semantic Compression: Spatial downsampling of input feature maps by convolutional operations to generate coarse-grained semantic anchors:

(8) $$X_{\mathrm{down}}=\mathrm{Conv}2D\left(X\right)\in R^{\frac{H}{k}\times \frac{W}{k}\times C}.$$

This procedure mimics rapid lesion localization at low magnification, compressing extraneous background areas while preserving the core morphological features of the lesion.

High-magnification multi-scale comparisons: Cross-scale contextual features are generated via pyramid vooling (PSP) in a compressed feature space. First, PSP is performed on the key and value to extract the multi-resolution semantics:

(9) $$K_{\mathrm{psp}}=\mathrm{Concat}\left(\mathrm{AvgPool}s_{i}\left(K\right)\right)s_{i}\in 1,2,4,16,\;V_{\mathrm{psp}}=\mathrm{Concat}\left(\mathrm{AvgPool}s_{i}\left(V\right)\right)s_{i}\in 1,2,4,16.$$

Multi-scale pyramid attention is then applied. The original spatial coordinates of the query vector (Q) are maintained, and fine-grained feature focusing is achieved by interacting with multi-scale key values:

(10) $$\mathrm{Attn}\left(Q,K_{\mathrm{psp}},V_{\mathrm{psp}}\right)=\mathrm{Softmax}\left(\frac{QK_{\mathrm{psp}}^{T}}{\sqrt{d}}\right)V_{\mathrm{psp}}.$$

This mechanism allows the model to simultaneously capture the overall distribution pattern of the lesion (s = 16) with microstructural details (s = 1), simulating the visual switching of a physician between different magnifications. In addition, this design transforms the multi-scale cognitive process of a doctor into a differentiable computational structure, providing a globally consistent and locally discriminative feature representation for subsequent hierarchical attention and forming a complete diagnostic simulation chain.

### 2.4. The Entire Network Structure

The whole network structure is shown in Figure 4. The input of the network is the original dermatoscopy image $I\in R^{H\times W\times 3}$. Subsequently, the multi-scale semantics are decoupled step-by-step through a four-level cascade of DCP-DHA-DFG processing units. At each level of processing, the DCP module performs spatial compression and cross-scale context fusion of the input features to generate low-resolution multi-granularity features. The DHA module then performs a two-phase route based on the multi-scale a priori output of the DCP. The background area is filtered through coarse region-level screening to filter the background region, after which token-level fine-grained attention is executed within the reserved region. The DFG module then performs channel decoupling and dynamic calibration on the attention output to suppress semantic contamination due to high-frequency noise such as hairs and bubbles. The final output is the lesion classification probability.

### 2.5. Experimental Configuration

To validate the performance of our dermatological classification model, we conducted extensive experiments on the publicly available ISIC 2018 and ISIC 2019 datasets [33].

The ISIC 2018 dataset is widely used for skin lesion analysis. It contains 10,015 images of skin lesions across seven different types: Actinic Keratoses and Intraepithelial Carcinoma (AKIEC), Basal Cell Carcinoma (BCC), Benign Keratosis (BKL), Dermatofibroma (DF), Melanocytic Nevi (NV), Melanoma (MEL), and Vascular Skin Lesions (VASC). The ISIC 2019 dataset is larger and more diverse, comprising 25,331 images spanning nine lesion categories. In this study we divided the datasets into training, validation, and test sets with a ratio of 0.8:0.05:0.15.

All experiments were conducted using the PyTorch (version 2.1.0) framework on a single NVIDIA RTX 4090 GPU with 24 GB of memory and running on a Linux system. To enhance model generalization [34,35], the data augmentation techniques of random flipping and random rotation were applied to the training set. Additionally, input images were normalized using a mean of [0.485, 0.456, 0.406] and standard deviation of [0.229, 0.224, 0.225] to maintain consistency in training.

The models were trained from scratch using a class-weighted cross-entropy loss function to address dataset imbalance [14]. Optimization was carried out with the AdamW optimizer. The initial learning rate was set to 0.0002, and a cosine annealing strategy was employed to gradually reduce the learning rate throughout the training process. Specifically, the learning rate decayed to 0.01 times its initial value within 100 epochs, ensuring smooth convergence towards an optimal solution. To mitigate overfitting, a weight decay parameter of 0.0002 was applied to constrain model complexity and enhance generalization performance. In order to balance efficient training with computational constraints, the batch size was set to 32 [36,37].

## 3. Results

### 3.1. Comparison Experiments

In this study, we systematically conducted comparative experiments to evaluate the performance of our proposed model against several classical CNNs (e.g., ResNet34, ConvNeXt) as well as the latest transformer networks (e.g.,ViT and SwinT). In addition, we compared DermViT with models that have performed well in recent years in the field of dermatology classification, such as RCSABC and Biformer. Table 1 and Table 2 present the experimental results of the different models on the ISIC2018 and ISIC2019 datasets. From the results of our comparative experiments, it can be observed that the proposed method outperforms other baseline models in terms of multiple key metrics while maintaining lower computational complexity and parameter count.

**Figure 5:**
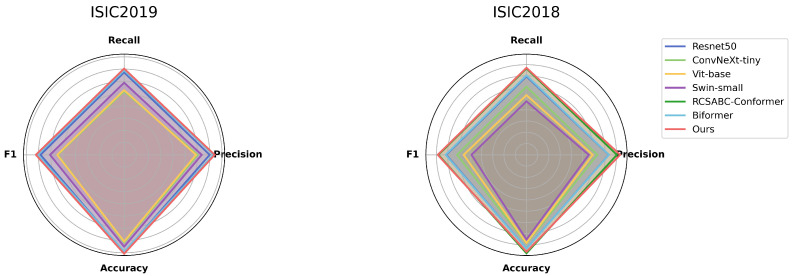
Radar charts for comparison experiments.

**Figure 6:**
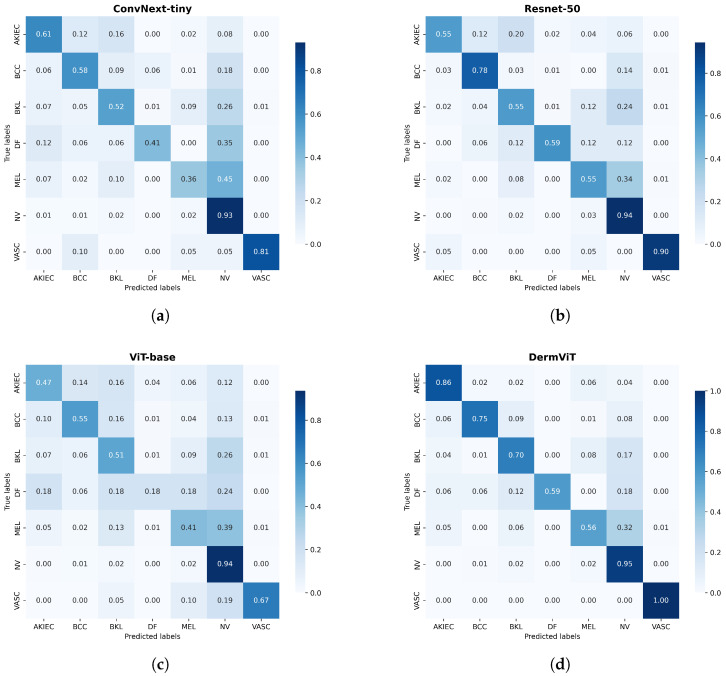
Multiclassification confusion matrices on the ISIC2018 dataset: (**a**) ConvNext-tiny model, (**b**) Resnet-50 model, (**c**) ViT-base model, and (**d**) our proposed DermViT model.

**Performance Analysis**: On the ISIC2018 dataset, DermViT outperforms benchmark models such as ResNet, ViT, SwinT, and ConvNeXt in terms of precision (80.75% ± 1.07%), recall (76.82% ± 0.50%), and MAUC (96.29% ± 0.05%). Although its F1-score (77.90% ± 0.43%) and accuracy (85.34% ± 0.23%) are slightly lower than those of RCSABC (78.3% and 87.39%, respectively), the differences are minimal, with RCSABC having a much larger parameter size (81.19 M vs. 16.82 M for DermViT). On the ISIC2019 dataset, DermViT maintains its lead in all evaluation metrics. The performance comparison is shown in Figure 5, where DermViT’s balanced results highlight its superior capability. As seen in Figure 6, DermViT also outperforms ConvNext-tiny, ResNet-50, and ViT-base in accuracy across all seven skin lesion categories, with only a slight performance gap in the BCC category.

Computational Efficiency Analysis: Our method significantly enhances computational efficiency, reducing FLOPs (2.75 G) and parameters (16.82 M) compared to ResNet50 (4.13 G FLOPs, 23.52 M parameters) and RCSABC (22.89 G FLOPs, 81.19 M parameters). This improvement stems from our redesigned DermViT block, which employs a more efficient attention mechanism for global feature modeling. Additionally, instead of traditional FFN layers, we use the convolution-based DFG module for feature transmission, which drastically reduces the number of parameters while preserving performance.

These results show that our model meets or even exceeds advanced models, with higher parameters in most of the key metrics, while still provides excellent classification results in resource-constrained application scenarios, verifying that our model can outperform traditional CNNs and transformers on the task of skin lesion classification while keeping computational and storage costs low. These results demonstrate DermViT’s combination of efficient parameter utilization and excellent classification performance.

### 3.2. Ablation Experiments

Based on the ablation experiment results shown in Table 3, we analyzed the impact of each proposed module on model performance and resource consumption.

The ViT model achieves 58.89% precision, 53.00% recall, 55.79% F1 score, 78.3% accuracy, and 94.1% on the MAUC metric, with FLOPS of 16.86 G and 85.65 M parameters.

Adding the DCP module increases the FLOPS to 5.12 G and the number of parameters to 32.36 M. Although the precision, recall, and F1 score all improve, the overall accuracy decreases slightly to 82.99% and the MAUC drops to 95.21%, indicating that while DCP enhances feature extraction, the increased parameter count impacts model efficiency.

Introducing the DFG module significantly reduces the FLOPS to 2.02 G and number of parameters to 11.52 M while improving precision to 75.4%, recall to 71.69%, and F1 to 73.37%. While overall accuracy drops slightly, MAUC remains at 95.4%, demonstrating that the DFG module maintains performance while reducing resource usage.

Finally, integrating all the modules results in FLOPS of 2.75 G and parameters of 16.82 M. Precision increases to 83.24%, recall reaches 77.25%, F1-score rises to 78.88%, accuracy improves to 86.12%, and MAUC reaches 96.54%. This combination shows a synergistic effect, significantly boosting classification accuracy and robustness while reducing resource consumption.

### 3.3. Training Process Analysis

**Figure 7:**
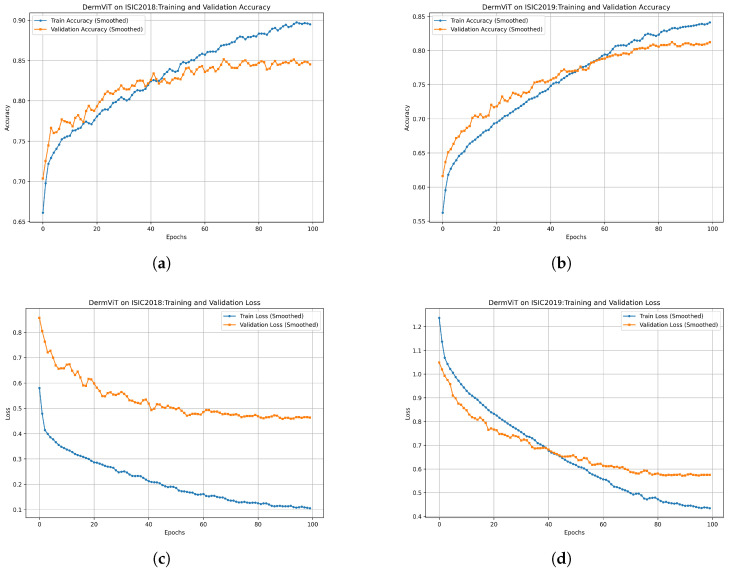
DermViT training and validation curves on the ISIC2018 and ISIC2019 datasets: (**a**) accuracy curves on ISIC2018, (**b**) accuracy curves on ISIC2019, (**c**) loss curves on ISIC2018, and (**d**) loss curves on ISIC2019.

In Figure 7, we present the training and validation process of the model on the ISIC2018 and ISIC2019 datasets, including accuracy curves and loss curves.

As shown in Figure 7a,b, the accuracy curves on both the ISIC2018 and ISIC2019 datasets exhibit an overall upward trend. The accuracy improvement on the ISIC2019 dataset is more stable, whereas the accuracy curve on the ISIC2018 dataset exhibits noticeable fluctuations. These fluctuations can be attributed to the smaller size of the ISIC2018 dataset leading to greater variance in training, whereas the larger ISIC2019 dataset contributes to a more stable training process.

In Figure 7c,d, it can be seen that the loss curves decrease rapidly at the beginning of training before gradually converging. The validation loss curve on the ISIC2019 dataset closely follows the training loss curve, indicating a more stable training process. In contrast, the gap between the training and validation loss curves is relatively larger on the ISIC2018 dataset, which may be due to the limited dataset size and lower sample diversity.

Overall, the four subfigures in Figure 7 demonstrate that the proposed model is able to effectively learn the features and converge on both the ISIC2018 and ISIC2019 datasets. The larger size of the ISIC2019 dataset contributes to a more stable training process, with the loss curves of the training and validation sets maintaining more consistent downward trends. Meanwhile, the fluctuations in the ISIC2018 curves reflect the impact of dataset size on the training process. These results further validate the effectiveness of the proposed method, and indicate that increasing the dataset size can enhance both training stability and generalization performance.

### 3.4. Visualization

**Figure 8:**
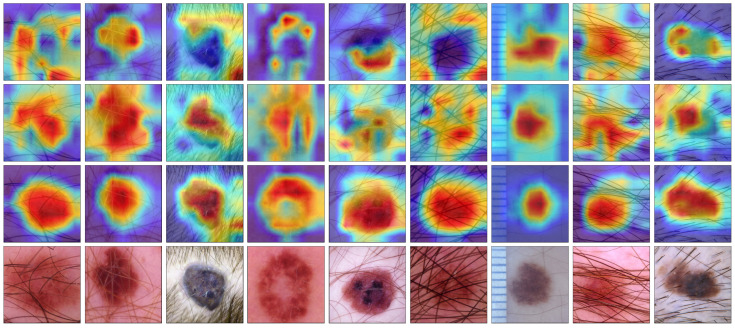
Grad-CAM visualization of images. In order from top to bottom, each line shows the results of ResNet50, Biformer, DermViT, and the original images.

The classification of skin lesion images often faces significant challenges due to artifacts such as hair and measurement tools, which can diminish the model’s attention and compromise diagnostic accuracy. To validate the clinical applicability of the DermViT model in scenarios with complex backgrounds, we conducted a visual analysis of lesion images containing typical interfering factors, as illustrated in Figure 8.

The experimental results demonstrate that DermViT exhibits an attention distribution that is highly consistent with dermatologists compared to the ResNet50 and Biformer models, with its heatmaps precisely covering the core lesion areas. Specifically, in melanoma samples with hair artifacts, the ResNet50 model’s attention is dispersed across the surrounding hair regions, whereas DermViT accurately focuses on the lesion area. This alignment with clinical diagnostic logic can be attributed to the synergistic mechanism formed by the DHA module’s hierarchical feature guidance, the DCP module’s cross-scale semantic association, and the gating mechanism of the DFG module.

The visualization results not only demonstrate the interpretability of the model’s decision-making process but also effectively validate the feasibility of modeling dermatologists’ diagnostic paradigms based on cognitive pathways.

## 4. Discussion

The DermViT model proposed in this paper achieves significant performance improvements in skin lesion classification tasks, validating the effectiveness of its medical-driven design. The success of DermViT highlights the importance of healthcare-driven architectural design in bridging the gap between technological advancements and clinical needs. By simulating the diagnostic workflow of dermatologists, DermViT not only achieves exceptional performance but also provides a more interpretable and trustworthy solution for skin lesion classification. This approach can be extended to other medical imaging tasks where domain-specific knowledge is crucial for accurate diagnosis, such as radiology and pathology [38].

**Figure 9:**
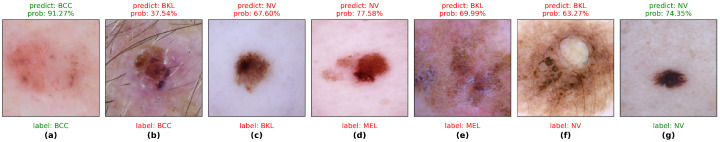
Example predictions of extreme cases from the ISIC2018 test set: (**a**) BCC (early), (**b**) BCC (late), (**c**) BKL (early), (**d**) MEL (early), (**e**) MEL (late), (**f**) NV (late), (**g**) NV (early).

Despite its notable advantages, DermViT still faces several challenges, particularly around addressing intra-class variability and inter-class similarity, which are both central issues in skin cancer diagnosis. Through an analysis of misclassified extreme cases from the test set (Figure 9), we identified specific limitations and potential areas for improvement.

Intra-Class Variability: The morphological characteristics of skin lesions such as melanomas can exhibit significant variations across different stages of progression. For example, early-stage BCC presents as a superficial lesion with indistinct borders, while late-stage BCC manifests as a nodular lesion with central ulceration. Although DermViT utilizes the DCP module to capture local texture information, it remains insufficiently sensitive to temporal morphological changes. The experimental results indicate that the model misclassified early- and late-stage BCC as distinct categories, highlighting its limited ability to model dynamic intra-class variations. This limitation is likely due to the model’s inability to account for the temporal evolution of lesions. In clinical practice, dermatologists often incorporate patient history to refine diagnoses. Thus, future research could explore integrating time series data or dynamic feature modeling in order to enhance DermViT’s ability to capture and adapt to intra-class variability.

Inter-Class Similarity: Inter-Class Similarity: The morphological overlap between different lesion categories (e.g., MEL and BKL) poses another significant challenge. For example, BKL and MEL both share visual characteristics that are similar to NV, such as clear border pigmentation, which leads DermViT to misclassify them as NV. The model appears to rely too heavily on superficial visual similarity, failing to distinguish the essential pathological differences between BKL, MEL, and NV. This suggests that DermViT may struggle to differentiate categories with similar visual features but distinct underlying pathological mechanisms, especially when it comes to identifying deeper pathological features such as cellular atypia. Enhancing the model’s ability to analyze this pathological substructure remains an ongoing challenge. Future work could explore higher-resolution feature encoding or incorporate pathological priors, which could improve the robustness of the model when handling such challenging cases.

Data Scarcity and Generalizability: While DermViT performs well on the ISIC datasets, its generalizability to other datasets and imaging modalities requires further validation. Future work could explore self-supervised or semi-supervised learning techniques [39,40] to further reduce reliance on large annotated datasets.

Computational Efficiency and Lightweight Design: Although DermViT demonstrates superior computational efficiency compared to other models, its parameter count needs further optimization in order to better adapt to mobile device deployment and enable efficient diagnosis in resource-constrained environments [41]. This is of significant practical value for regions with limited access to healthcare services [42].

Interpretability: While the attention mechanism in DermViT provides a degree of interpretability, more advanced tools could be developed to enhance clinicians’ trust in the model [43]. Techniques such as saliency maps or explainable AI frameworks could be integrated to improve transparency [44].

Multimodal Fusion: Integrating additional data sources such as patient metadata or genomic data could further enhance diagnostic accuracy and enable personalized treatment recommendations. Multimodal approaches have shown promise in improving the robustness and applicability of medical AI systems [45,46].

By addressing these challenges, DermViT and similar models can continue to advance the field of medical imaging, offering more reliable, accessible, and interpretable diagnostic solutions for diverse clinical applications.

## 5. Conclusions

This paper presents DermViT, a medically-driven vision transformer tailored for skin lesion classification. DermViT tackles key challenges such as semantic entanglement, multi-scale feature variations, and computational inefficiency through three novel modules (DCP, DFG, and DHA) that mimic the diagnostic processes of dermatologists. This design enhances performance, interpretability, and robustness, making for significantly reduced misclassification against complex backgrounds. Experiments on the ISIC2018 and ISIC2019 datasets validate the effectiveness of DermViT, reaching 86.12% accuracy on ISIC2018 (a 7.8% improvement over ViT-Base) and strong generalization on ISIC2019. Additionally, DermViT maintains high accuracy while reducing the number of parameters compared to ViT, enhancing its suitability for real-time diagnosis in resource-limited settings. By integrating medical expertise with deep learning, DermViT offers a reliable high-performance solution for skin lesion classification.

## Figures and Tables

**Table 1:** Experimental results on the ISIC2018 dataset.

Model	FLOPs (G)	Parameters (M)	ISIC2018				
			Precision (%)	Recall (%)	F1 (%)	Accuracy (%)	MAUC (%)
Resnet34	3.68	21.8	69.02 ± 2.38	66.80 ± 0.90	67.24 ± 1.42	81.67 ± 0.52	95.56 ± 0.20
Resnet50	4.13	23.52	71.33 ± 1.04	69.58 ± 1.60	70.14 ± 0.94	82.35 ± 0.46	95.58 ± 0.17
Resnet101	7.87	44.55	68.92 ± 1.77	69.20 ± 1.36	68.67 ± 0.92	81.93 ± 0.41	95.68 ± 0.11
ConvNeXt-tiny	4.45	27.8	61.50 ± 1.16	59.05 ± 1.76	59.18 ± 1.33	78.09 ± 0.33	94.27 ± 0.07
Swin-tiny	8.74	27.5	62.28 ± 0.95	62.41 ± 1.14	61.88 ± 1.11	80.04 ± 0.25	95.42 ± 0.04
Swin-small	17.09	48.8	52.80 ± 0.64	47.63 ± 0.73	49.70 ± 0.51	74.11 ± 0.27	92.80 ± 0.04
ViT-Base	16.86	85.65	60.86 ± 2.22	55.03 ± 1.38	56.88 ± 1.50	77.75 ± 0.42	93.00 ± 0.21
RCSABC [19]	22.89	81.19	80.38	76.66	**78.3**	**87.39**	\
Biformer	4.42	27.65	71.17 ± 1.10	71.44 ± 1.49	69.86 ± 0.97	83.00 ± 0.45	95.18 ± 0.13
DermViT	**2.75**	**16.82**	**80.75** ± 1.07	**76.82** ± 0.50	77.90 ± 0.43	85.34 ± 0.23	**96.29** ± 0.05

Note: Bolded data is the best under this indicator, and underlined data indicates second place.

**Table 2:** Experimental results on the ISIC2019 dataset.

Model	FLOPs (G)	Parameters (M)	ISIC2019				
			Precision (%)	Recall (%)	F1 (%)	Accuracy (%)	MAUC (%)
Resnet34	3.68	21.8	68.42 ± 0.93	66.59 ± 0.91	67.11 ± 0.87	78.92 ± 0.46	95.75 ± 0.06
Resnet50	4.13	23.52	70.31 ± 0.86	68.00 ± 0.43	68.83 ± 0.51	79.38 ± 0.21	96.07 ± 0.06
Resnet101	7.87	44.55	66.47 ± 0.73	65.49 ± 0.88	65.57 ± 0.61	77.44 ± 0.34	95.41 ± 0.07
ConvNeXt-tiny	4.45	27.8	58.90 ± 1.21	51.71 ± 1.50	53.94 ± 0.93	71.55 ± 0.27	93.21 ± 0.05
swin-tiny	8.74	27.5	56.96 ± 1.40	42.23 ± 1.39	45.17 ± 1.36	67.48 ± 1.19	89.79 ± 0.23
swin-small	17.09	48.8	63.40 ± 0.38	58.53 ± 0.37	60.43 ± 0.15	74.80 ± 0.12	93.89 ± 0.02
Vit-base	16.86	85.65	58.66 ± 0.52	52.61 ± 0.90	54.54 ± 0.78	70.40 ± 0.33	92.39 ± 0.07
RCSABC [19]	22.89	81.19	\	\	\	\	\
Biformer	4.42	27.65	72.35 ± 1.04	69.52 ± 1.17	70.68 ± 0.75	78.77 ± 0.31	95.71 ± 0.12
Ours	**2.75**	**16.82**	**72.39** ± 0.41	**71.24** ± 0.31	**71.57** ± 0.30	**80.82** ± 0.20	**96.42** ± 0.03

Note: Bolded data is the best under this indicator, and underlined data indicates second place.

**Table 3:** Ablation experiment results on the ISIC dataset.

Model	FLOPs	Parameters (M)	Precision (%)	Recall (%)	F1 (%)	Accuracy (%)	MAUC
Baseline	16.86	85.65	58.89	53.00	55.79	78.3	94.01
Baseline + DHA	4.42	27.65	73.34	70.50	71.89	83.26	96.02
Baseline + DCP	5.12	32.36	74.76	71.18	72.93	82.99	95.21
Baseline + DFG	2.02	11.52	75.40	71.69	73.37	82.52	95.40
Baseline + DHA + DCP + DFG	**2.75**	**16.82**	**83.24**	**77.25**	**78.88**	**86.12**	**96.54**

Note: Bolded data is the best under this indicator.

## Data Availability

The original data presented in the study are openly available in the International Skin Imaging Collaboration (ISIC) challenges at https://challenge.isic-archive.com/landing/2018/ (accessed on 2 May 2024) and https://challenge.isic-archive.com/landing/2019/ (accessed on 5 May 2024). The code will be available in the future at https://github.com/llyafei/DermViT (accessed on 1 April 2025).
